# Qudi-HiM: an open-source acquisition software package for highly multiplexed sequential and combinatorial optical imaging

**DOI:** 10.12688/openreseurope.14641.2

**Published:** 2022-07-19

**Authors:** Franziska Barho, Jean-Bernard Fiche, Marion Bardou, Olivier Messina, Alexandre Martiniere, Christophe Houbron, Marcelo Nollmann

**Affiliations:** 1 Centre de Biologie Structurale, Centre National de la Recherche Scientifique, UMR5048, Montpellier, 34090, France; 2 Institut Agro Montpellier, INRAE, Montpellier, 34000, France

**Keywords:** optical microscopy, multiplexed imaging, chromosome organization, transcription

## Abstract

Multiplexed sequential and combinatorial imaging enables the simultaneous detection of multiple biological molecules, *e.g.* proteins, DNA, or RNA, enabling single-cell spatial multi-omics measurements at sub-cellular resolution. Recently, we designed a multiplexed imaging approach (Hi-M) to study the spatial organization of chromatin in single cells. In order to enable Hi-M sequential imaging on custom microscope setups, we developed Qudi-HiM, a modular software package written in Python 3. Qudi-HiM contains modules to automate the robust acquisition of thousands of three-dimensional multicolor microscopy images, the handling of microfluidics devices, and the remote monitoring of ongoing acquisitions and real-time analysis. In addition, Qudi-HiM can be used as a stand-alone tool for other imaging modalities.

## Motivation and significance

**Figure 1.**
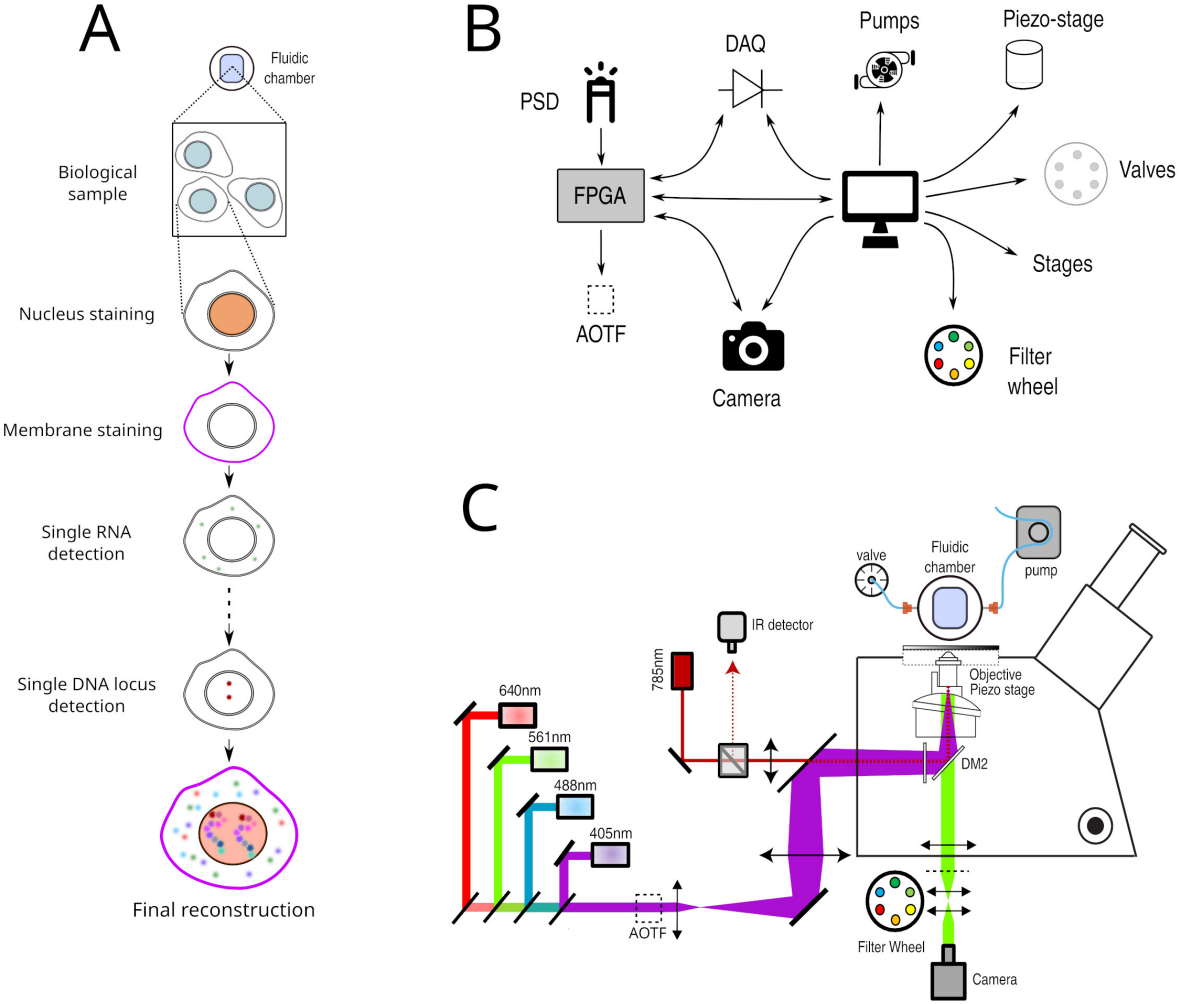
**A**. Principle of Hi-M imaging. A single region of interest within the biological sample contains several cells. In cycle 0, a DNA specific dye is used to label the nucleus and other markers. In the following cycles, different probes are injected and imaged. Data from all cycles are combined to reconstruct the final image. **B**. Overview of hardware devices that are addressed by Qudi-HiM. **C**. Schematic drawing of a Hi-M microscope. The setup shown here includes 4 excitation lasers (405, 488, 561, 640 nm), an acousto-optic tunable filter, flat and dichroic mirrors, a microscope objective, emission filters, microscope camera, a 785 nm infrared laser and detector, sample holder with microfluidic chamber, sample positioning stage, and a liquid handling robot.

Cutting-edge developments of novel fluorescence microscopy methods have flourished over the last decade, particularly those aimed at revealing the organization and dynamics of biological systems at multiple scales ^1– 3^. These developments focused particularly on critical technological limitations, such as spatial resolution, imaging depth, imaging speed, or photo-toxicity ^4^. An additional inherent limitation of fluorescence microscopy methods relies on their ability to image multiple molecular species at once. Typically, the restriction arises from the overlap in the emission spectra of available fluorophores. Recently, this limitation was addressed by several technologies that combine liquid-handling robots to widefield fluorescence microscopy to perform sequential or combinatorial acquisition of different molecular species, such as RNA, DNA or proteins ^5– 13^ ( Figure 1A). These multiplexing imaging technologies now enable the detection of hundreds to thousands of different biological objects at once ^14, 15^.

The simultaneous detection of multiple targets is particularly important to understand the organization and function of chromatin at the single-cell level ^9– 12^. In eukaryotes, chromatin is organized at multiple length scales, from nucleosomes to chromosome territories ^16^. Multi-scale three-dimensional (3D) conformation is involved and influences many biological processes, such as transcription ^17^. Thus, it is of particular importance to be able to simultaneously visualize 3D chromatin organization and transcriptional output in single cells.

We recently developed a multiplexed imaging approach (Hi-M) to simultaneously capture the conformation of chromatin and the transcription of genes in single cells ^5, 10^, and used it to investigate whether and how single-cell chromatin structure influences transcription ^18^. In Hi-M, as in the other multiplexing methods mentioned above, each cycle of acquisition requires the coordinated control of several fluid-handling hardware components ( *e.g.* pumps, valves, motorized stages) to deliver and incubate different fluids into the sample, and of multiple devices ( *e.g.* acousto-optic tunable filters, piezoelectric and motor stages, cameras) to acquire 3D images at multiple regions of interest (ROIs) ( Figures 1B, 1C). Acquisition of each of these sequential cycles can require tens of minutes, thus a typical Hi-M experiment with tens of cycles can last several days of continuous and unsupervised acquisition (depending on sample and number of ROIs). As a consequence, automatic, flexible, and robust experimental control software is required for efficient and reproducible multiplexed measurements.

Here, we present Qudi-HiM: an open-source, modular, user-friendly and robust acquisition interface written in Python 3 to perform sequential and high-throughput fluorescence imaging. The software was developed with a focus on multiplexed imaging of DNA, RNA and proteins, but can be easily extended to other fluorescence imaging modalities such as single-molecule localization microscopy ^19^.

## Software description

### Software architecture

***Python software packages, a popular alternative to commercial environments*.** Developing custom software is often necessary when implementing new microscopy technology, as this provides enough flexibility for interfacing different hardware components and optimizing custom acquisition workflows. Common approaches used to develop custom software include the use of commercial environments, such as LabView (National Instruments, US) (RRID:SCR_014325), that can interface with APIs (application programming interfaces) or SDKs (software development kits) to control hardware. However, LabView requires expensive licenses, depends on upstream development from the hardware constructors, and is less adaptable to version control systems compared to textual programming languages due to its binary content.

As an alternative, we turned to Python (RRID:SCR_008394), a popular programming language that is intelligible due to its high level of abstraction and that benefits from a dynamic and expanding scientific user community ^20^. This is further illustrated by the fact that most hardware components can now be interfaced using Python packages, either developed by the constructors or end-users, and are often freely available on GitHub. A large set of Python packages are continuously developed for image analysis, for graphical user interface design and for hardware control. For example, the majority of novel image analysis involving artificial intelligence methods typically provide Python APIs ^21– 23^. Finally, Python can directly interface with SDK libraries to control hardware devices and offers the possibility to combine data acquisition and real-time analysis using Python APIs from robust, community-led packages ( *e.g.* NumPy (RRID:SCR_008633) ^20^, scikit-image (RRID:SCR_021142) ^23^ or astropy (RRID:SCR_018148) ^24, 25^) or from newly developed image analysis methods. Python is therefore an appealing choice for designing home-build microscopy control software.

Several software packages have been recently developed using Python for various imaging modalities. These include Qudi ^26^, the software package Qudi-HiM is based on; Python Microscopy; Oxford Microscope-Cockpit ^27^; Tormenta ^28^; Storm-Control; and MicroMator ^29^. 

***Qudi*.** Qudi is a modular laboratory experiment management suite written in Python 3 and the Qt Python-binding pyQt. It provides a stable and comprehensive framework for the development of custom graphical user interfaces (GUI) and hardware interfacing. The Qudi architecture consists of a core infrastructure that envelopes application-specific science modules.

Qudi’s core contains the general functionalities for program execution, logging, error handling and configuration reading. Its structure is not limited to a particular scientific application. Hence, it was possible to reuse it as the backbone for Qudi-HiM ^30^. Its central part is a manager module that uses a setup-specific configuration file to load and connect hardware components and control them through so-called science modules.

The science modules provide all the functionalities that are required for the control of a specific instrument. These modules are organized into three categories following the model-view-controller design pattern: hardware modules, experimental logic, and GUI. Hardware modules represent the physical devices and make the basic hardware functionalities accessible. The experimental logic assembles hardware commands into higher-level operations to control experiments in a convenient manner. Experimental logic modules serve as a communication channel between GUI and hardware. Due to the use of an abstract parent class for hardware devices that provide the same functionalities ( *e.g.* different camera technologies or different types of actuators for controlling sample position), interchanging hardware modules is completely transparent for the experimental logic layer. Finally, the GUI provides a means to control the experiments and to directly visualize data without an in-depth knowledge of the underlying code. This software architecture enables rapid adaptation and portability to microscope setups with different hardware components, and facilitates long-term maintenance, adaptation to new user needs, and development of new functionalities. Finally, the use of the same GUI and underlying logic for different microscopy setups ( *e.g.* widefield, time-lapse or Hi-M imaging) simplifies user training, which is advantageous for imaging facilities open to multiple users with different backgrounds.

### Software functionalities

Multiplexed imaging requires not only the selection and synchronization of light sources, filters, detectors and the control of sample displacements, but also the handling of injection of liquids by a robotic system consisting of valves, pumps, and motorized stages ( Figures 1B, 1C). To decouple these different operations and to make the acquisition of Hi-M datasets user-friendly and flexible, we developed separate modules for imaging, sample positioning and for fluidics handling. The GUI modules and their main functionality are summarized in Table 1 and described in the following sections.

**Table 1.** Overview of Qudi-HiM graphical user interface (GUI) modules with their key functionalities. The corresponding graphical user interfaces are shown in Figure 2 and Figure 3. Colorcode: yellow – imaging modules, light red – fluidics modules, blue – automation modules.

GUI Module	Functionality
Basic imaging toolbox	Real-time acquisition Camera settings and status Illumination control (laser & white light source) Filter control
Region of interest (ROI) selector	Spatial position navigation Registration of ROI lists Mosaic and interpolation tools
Focus toolbox	Manual focus Autofocus calibration Focus stabilization mode Local focus search
Fluidics toolbox	Valve position selector 3-axes positioning stage for probe selection Pressure control, flow rate and volume measurement
Injections configurator	Definition of injection sequences for Hi-M experiments
Experiment configurator	Variable form for easy configuration of various experiment types

### Basic tools for imaging

**Figure 2.**
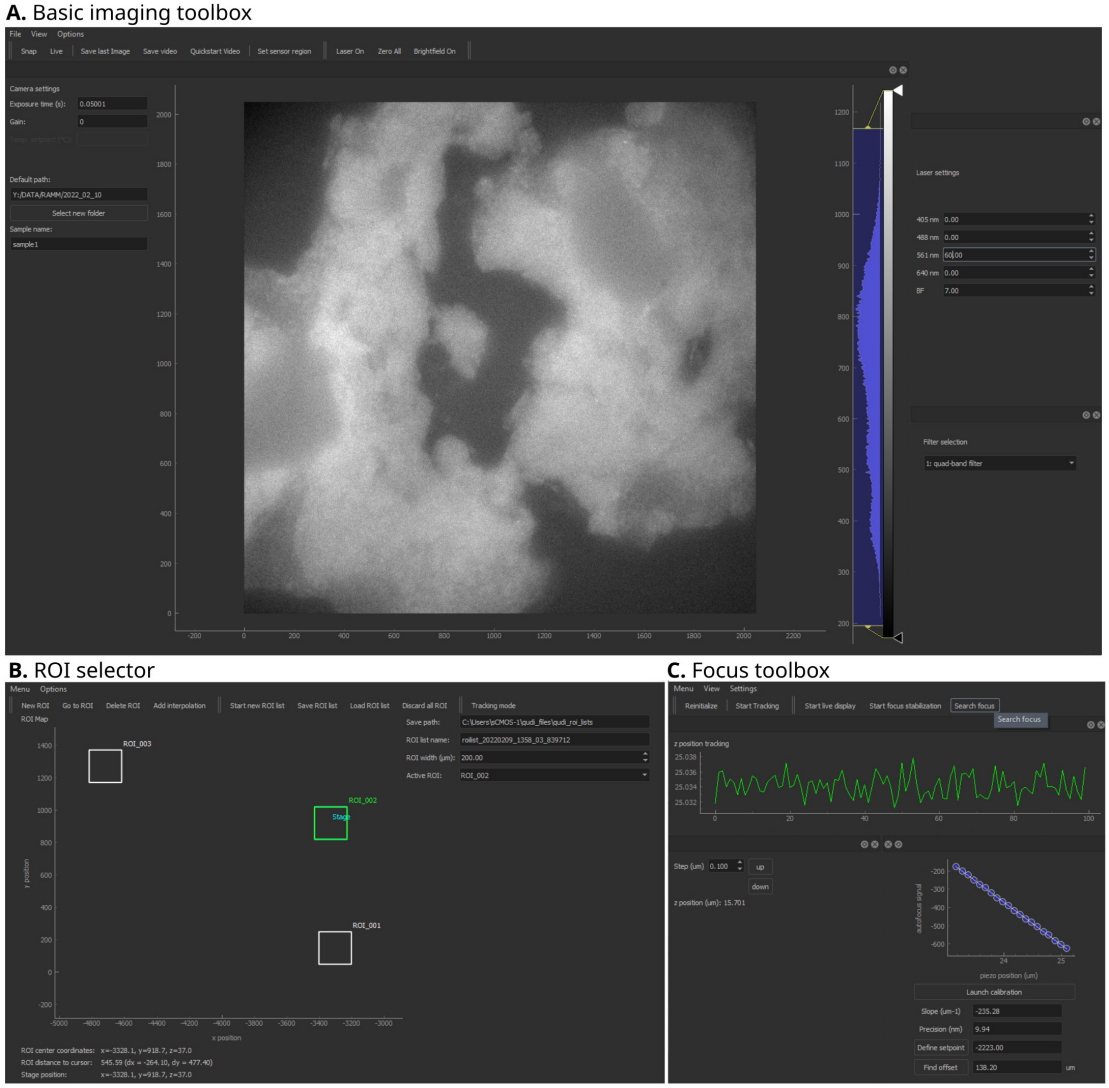
Qudi-HiM imaging graphical user interface (GUI) modules. **A**. Basic imaging toolbox. **B**. Region of interest (ROI) selector. **C**. Focus toolbox. Key functionalities of each module are summarized in Table 1.

***Basic imaging toolbox*.** The Basic imaging module is a polyvalent tool for custom microscopes that provides all fundamental operations needed for imaging on an intuitive user interface ( Figure 2A). In the context of multiplexed imaging, this includes the visual inspection of the sample to assess its quality using fluorescence and brightfield imaging, such as checking for labeling efficiency and specificity, cell density, tissue integrity, *etc*. Specifically, this module allows for the selection of intensity and wavelength of the light sources, the switching of filters, and the setting of acquisition parameters for the camera, such as exposure time, gain, acquisition mode, and sensor temperature, independently of the camera technology (emCCD, sCMOS, CMOS) deployed on a setup. Besides real-time inspection, imaging data can be saved using multiple common formats such as TIFF, FITS ( FITS Documentation Page) or NumPy arrays ^20^.

***ROI selector*.** Multiplexed microscopy involves the repeated acquisition of images in several regions of interest per cycle. Hence, a module was developed to manually or automatically select the ROIs that will be acquired during a multiplexed acquisition ( Figure 2B). Automatic selection of ROIs includes mosaic and interpolation tools to explore larger portions of a sample efficiently using snake-like patterns. Mosaics are constructed by defining the central position, and the number of tiles. Alternatively, the user can define the edges of a region to be explored and the module will automatically interpolate tiles to cover the entire region. For both automatic ROI selection methods, an overlap area between tiles can be parametrized to optimize tile realignment and reassembly.

***Focus toolbox*.** The Focus module ( Figure 2C) is used to change the focal plane by translating the objective or sample relative to each other by means of a piezo-electric or motorized stage. In addition, this module maintains the selected focal plane by using a feedback system. The autofocus system uses the reflection of a near-infrared laser (785 nm) to detect changes in the objective-sample distance ^31^ and actively maintains the setpoint using a proportional-integral-differential (PID) controller. Thus, depending on the sample and the requirements of each experiment, this module can either be used to maintain focus over time or to locally define the focal plane for each ROI. For both applications, several modalities have been developed depending on the detectors available on the microscope ( *e.g.* CMOS camera, position sensitive device, *etc*.).

**Figure 3.**
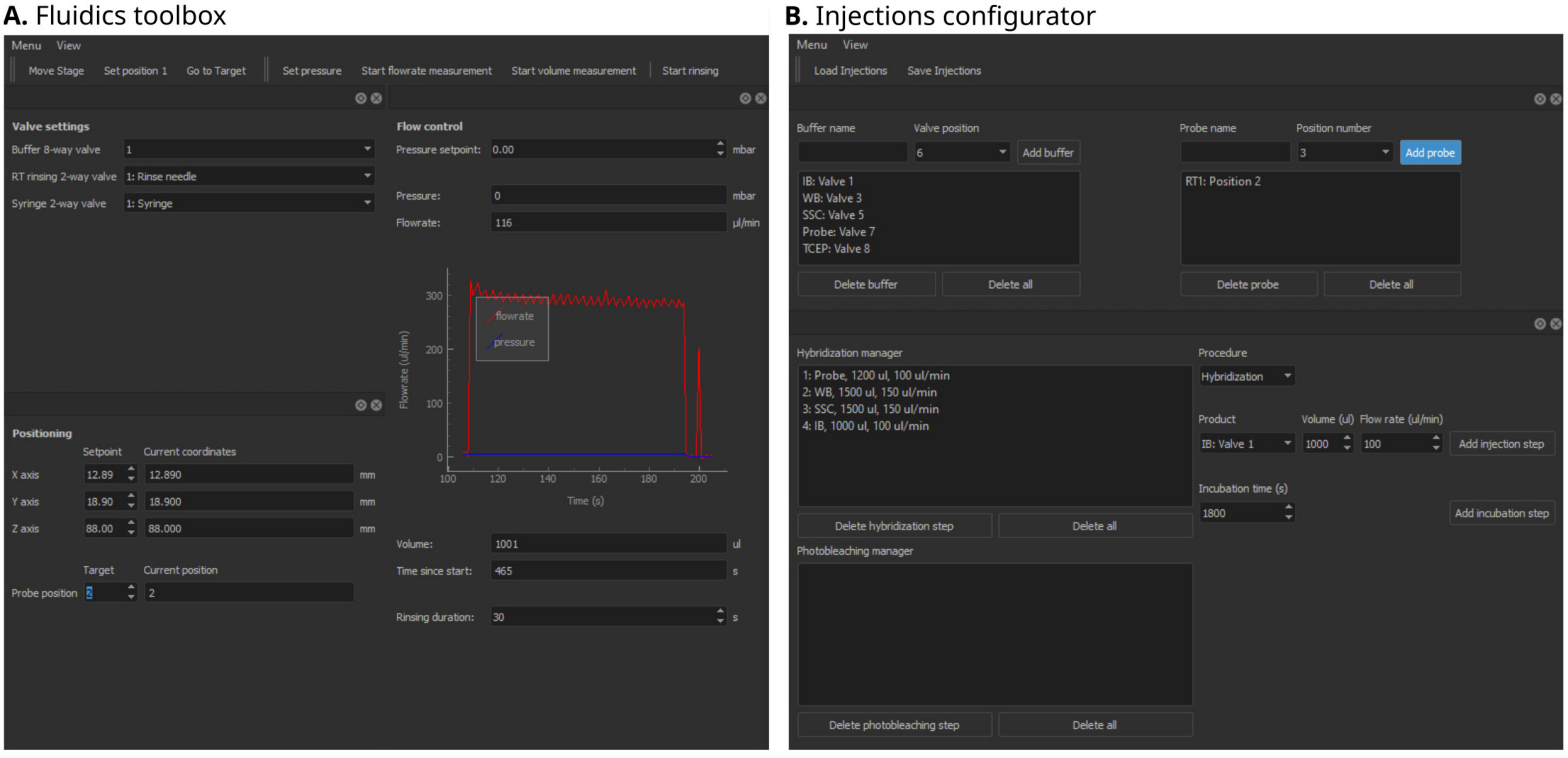
Qudi-HiM fluidics graphical user interface (GUI) modules. **A**. Fluidics toolbox. **B**. Injections configurator. Key functionalities of each module are summarized in Table 1.

***Fluidics toolbox*.** The purpose of the fluidics GUI ( Figure 3A) is to control the devices that deliver different liquid solutions into the microfluidics chamber. The hardware components include modular valve positioners, pumps, flow-rate sensors, as well as motorized stages, which together form a custom-made liquid handling and delivery robot. Using these devices and the operations defined in the underlying experimental logic, injection sequences can be performed in a controlled and reproducible manner. Fine tuning of the flow rate and injected volumes is achieved using a PID feedback loop on the pump system.

***Injections configurator*.** A sequential acquisition experiment requires repeating a series of injection and incubation steps in a well-controlled manner that can include: hybridization of imaging probes, washing steps to remove unbound probes, chemical bleaching, and introduction of buffers to prevent photobleaching during imaging. Qudi-HiM provides a GUI ( Figure 3B) to configure injection sequences, including information on the products ( *e.g.* names, valve positions, etc.), the target volume, required flow rate and eventually, incubation time.

### Automatized experiments

Qudi natively provides a handler for iterative experiments: the Task runner. This module is based on a state machine that handles a sequence of steps such as ‘starting’, ‘running’, ‘pausing’, ‘resuming’, ‘finishing’, ‘stopped’, *etc*. Custom experiments, called “Tasks”, are defined by the actions performed on each state transition.

Several modules of a typical Hi-M experiment ( *e.g.* acquisition of multicolor stacks of images, injection sequences, *etc*.) as well as the complete pipeline for a Hi-M acquisition were implemented and made available in the Task runner. The idea behind this implementation was to turn Qudi-HiM into a versatile imaging tool: efficient for running complex experiments such as Hi-M and helpful for executing simpler acquisitions, such as those required during experiment setup, protocol optimization or exploratory tests.

**Figure 4.**
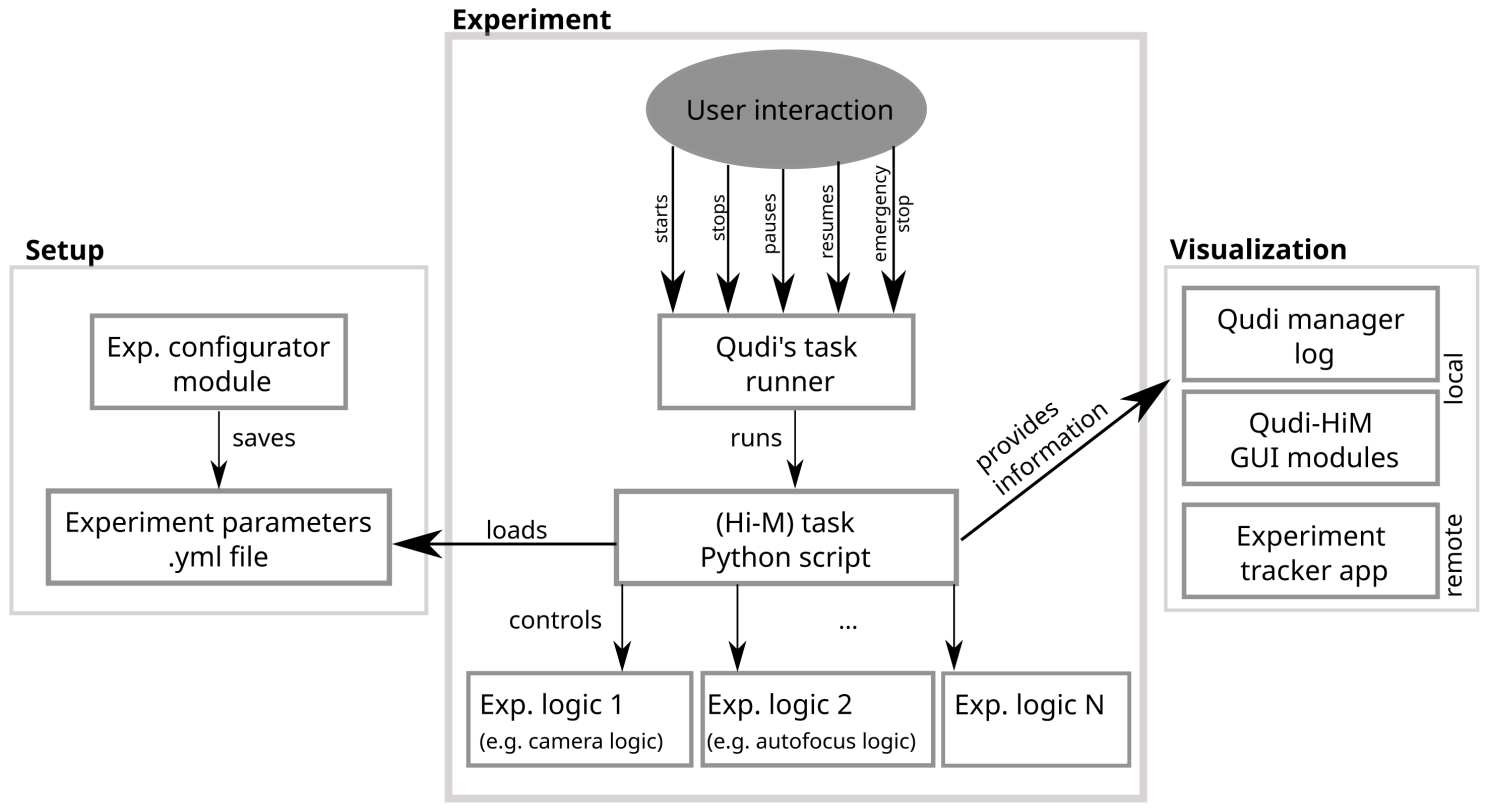
Scheme of the workflow used by Qudi-HiM to set up, run and control automatized experiments.

Each task requires a set of user parameters that needs to be easily accessible and adapted according to the sample and the experimental conditions. For this reason, an experiment configurator module for the setup of experiments is included in Qudi-HiM. Depending on the selected task, the configurator GUI displays a form that provides access to all required parameters and verifies their validity before saving the configuration ( Figure 4). The configuration is stored as a YAML file and is subsequently loaded by the Python script that contains all information for the task execution. The script itself is controlled via the Task runner GUI: Here, the user selects which task to run and has the ability to run, stop, pause or resume the task. The task script connects to all required experimental logic modules to combine the necessary high level hardware actions.

Since multiplexed acquisitions can last for several days, it is convenient for the users to track the current status of their experiment. Besides visualization on the Qudi-HiM GUIs and a textual information status on the log for local observation, we also provided a file exchange-based interface from Qudi-HiM software to a status-tracker application using Bokeh ( Bokeh documentation) to display key parameters of the experiment (e.g. cycle number, fluidics status, etc.) and a real-time pre-analysis of the acquired image data.

### Implementation of hardware dummies

Qudi-HiM may be used on a computer without connected devices thanks to the implementation of dummy devices: mock-ups of the microscope hardware components that simulate their physical response. The dummies are especially useful during development and for the initial tests of the logic and GUIs. They were introduced in the Qudi software package and we consistently continued developing this approach, including the use of dummy tasks to simulate sequences of the Hi-M experiment for optimization

### Currently supported hardware and extensions

An overview of all devices that are available in the current version of Qudi-HiM is shown in Table 2. An online resource with the most up to date supported hardware is available here. Swapping devices between experimental setups is straight-forward, as long as the device has been interfaced in Qudi-HiM’s hardware modules. This simply requires updating the setup configuration file to interface the new piece of hardware. Example entries are provided in the hardware modules documentation (see examples of configuration files at: https://github.com/NollmannLab/qudi-HiM/tree/master/config/custom_config). 

**Table 2.** Overview of currently available hardware in Qudi-HiM.

Hardware class	Supported devices
Camera	Andor emCCD - iXon Ultra 897 Hamamatsu sCMOS - ORCA-Flash4.0 V3 Thorlabs CMOS - DCC1545M
DAQ	National Instruments DAQ - M-Series PCIe-6259 Measurement computing DAQ - USB3104
FPGA	National Instruments RIO FPGA - PCIe-7841R
Lightsources	Lumencor Celesta Light Engine
Motorized stages	Applied Scientific Instrumentation - MS-2000 XY and XYZ Physik Instrumente Motor - Controller C-863 Mercury and C-867
Single axis piezoelectric stage for objective	Physik Instrumente - E-625 Controller & PIFOC stage Mad City Labs - NanoDrive & Nano-F-100
Filter wheels	Thorlabs - FW-102C Thorlabs - FW-103 High-Speed Motorized & APT Motor BSC201
Microfluidics	Fluigent - Flowboard FLB Fluigent Pump - MFCS-EZ Fluigent Flow Rate Sensor - Flow Unit L Hamilton Modular Valve Positioner

Extending the available hardware is achieved by setting up a new hardware module and implementing an abstract interface using existing Python APIs or C libraries. Finally, the configuration file is updated to interface the new hardware component.

## Use cases

**Figure 5.**
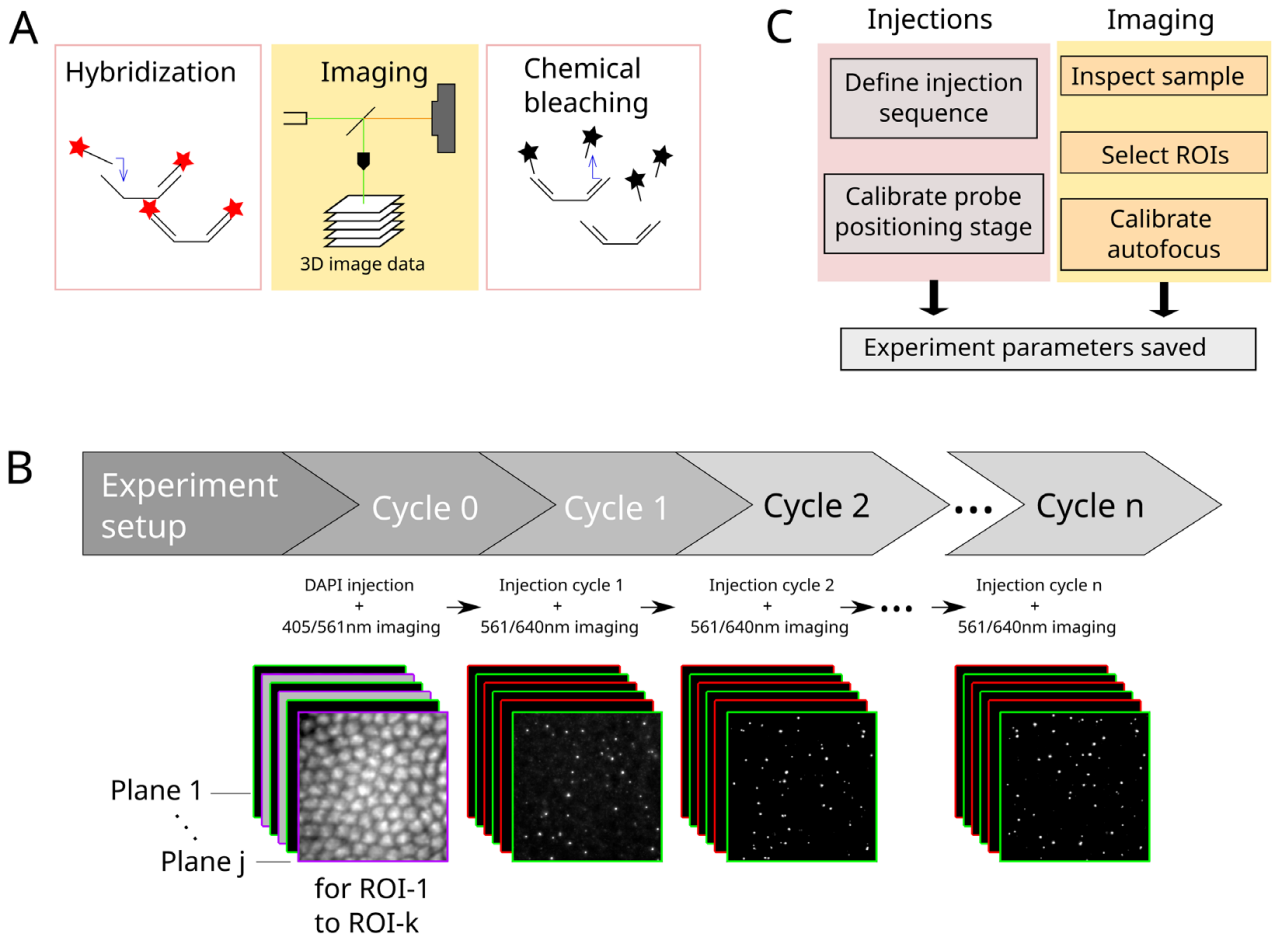
**A.** Each cycle in a Hi-M experiment consists of the hybridization with imaging probes, image data acquisition and subsequent chemical bleaching. **B**. Process flow of a typical Hi-M experiment. Multiple-color 3D images are acquired in each cycle, for multiple ROIs. From cycle 1 on, the experiment runs autonomously. **C**. Overview of steps performed during experiment preparation for subsequent automatized fluidics handling and imaging.

A typical Hi-M experiment requires a succession of 20–60 hybridization / image acquisition / chemical bleaching cycles ( Figure 5A). In each cycle, 3D multicolor image data are acquired on multiple predefined ROIs ( Figure 5B). Critical steps to ensure the success of the experiment are: (1) the precise control of injected volumes at target flow rates for each step; (2) the correct and reproducible focusing of the sample for all the pre-defined ROIs (see Refs 5, 10 for a detailed description of the autofocus hardware); (3) the reliable synchronization between light sources, camera and actuators controlling the sample XYZ position; (4) the storage of imaging data using a filename format encoding the cycle number and ROI, and of metadata containing the imaging and fluidics parameters. For Hi-M experiments, metadata saved with each image include sample name, imaging information such as exposure time, laser lines and intensity, autofocus parameters, sample XY position, scan step size and total Z range covered. To comply with a standardized metadata format ^32^ depending on the type of experiment that is performed, it is possible to add custom information by adapting the underlying software module containing the instructions of the experiment.

A Hi-M experiment starts with the injection of a DNA-specific dye to identify each single cell and fiducial imaging probe used for later image registration ^5, 10^. This procedure, designated by cycle 0 in Figure 5B, is performed separately from the other cycles since it requires a very specific combination of injections and incubation periods. The user first defines the adequate procedure using the injection configurator ( Figure 3B): for each single step the user chooses which solution to inject, the target volume and the expected flow rate (typically 1–2 ml at 150–300 µl/min). Then, the automatic procedure is launched using a dedicated fluidics task from the Qudi Taskrunner. During this procedure, the program continuously monitors the flow-rate according to the user’s requirements and adjusts the pump speed through a PID controller. When the right amount of solution is injected, the program automatically switches to the following solution or incubation period until the procedure is completed. Next, the user checks the quality of the fiducial labeling and if the cells have been properly labelled using the imaging toolbox ( Figure 2A, continuous illumination with either 405 nm or 561 nm laser). Finally, the ROIs are manually selected using the ROI selector ( Figure 2B), by identifying the most relevant sample positions. These ROIs positions are saved as a YAML file.

In anticipation of the successive acquisition cycles and to ensure proper reproducibility, a focus calibration is performed using the Focus toolbox GUI ( Figure 2C, Figure 5C). The user selects a typical ROI, focuses on the sample and launches the calibration procedure: the program performs a quick 2 µm ramp of the objective axial position while monitoring the position of the 785 nm laser beam thanks to a quadrant photodiode. If the calibration is valid (monotonous displacement with a precision <30 nm), the user defines the reference position of the 3D stack by adjusting the position of the objective lens in relation to an axial piezo stage. A starting position 1-2 µm below the sample focal plane is usually chosen, to prevent the acquisition of incomplete 3D data due to variability in the axial position of the biological sample.

Finally, a multi-color stack of images is acquired for each ROI using a Hi-M sub-task developed for this purpose. For this step, the user sets the imaging conditions using the experiment configurator (laser lines, laser intensity, stack size, etc.) and indicates where to find the ROI YAML file. For a standard experiment, 20–40 ROIs are selected, and for each of them a stack of 60–70 planes, each separated by 250 nm, are acquired simultaneously in two or three colors with 50 ms exposure. For each ROI, the program launches a 3D-stack acquisition by synchronizing the camera exposure with the lasers and the piezo displacements. Following each acquisition, the images are saved and the program moves to the next ROIs, in the order indicated by the user.

Following this initial cycle, the injection sequences for the Hi-M probes are defined ( Figure 5C). First, buffers common to all cycles are connected to a specific valve outlet and its position is entered in the injections configurator GUI ( Figure 3B). Second, cycle-specific Hi-M probes are arranged on the delivery tray of the pipetting robot by the user and their names and positions are entered into the GUI, according to their injection order. Third, the injection sequences for the hybridization and the chemical bleaching procedure are indicated for each single step, the user chooses which solution to inject, the target volume and the expected flow rate. After each injection, an incubation time can be further added if required. Finally, the complete injection procedure is saved as a YAML file and can hence be reused for similar experiments.

The final configuration step involves setting the acquisition parameters using the experiment configurator ( Figure 5B, cycles 1 to n). While the experiment is running, concurrent actions that could be triggered via the user interfaces are disabled. Both an emergency stop as well as a smooth transition to stopping or pausing the experiment after the current cycle are supported. The current status of the experiment can be tracked in real-time using the log displayed by the Qudi manager module. Relevant information ( *e.g.* flow rate, ROI position, etc.) are also displayed on the GUI elements that remain accessible during an experiment.

Additionally, the experiment can be followed remotely using the status tracker application that displays the most important information of the experiment in real time as well as real-time image analysis (see section Automatized experiments above).

## Conclusions

Qudi-HiM provides a range of functionalities from essential toolkits for live imaging up to fully automated experiments with a major focus on multiplexed imaging. For the latter, optical imaging is coordinated with microfluidic injections. Hence, all the necessary tools to control microfluidics and home-build microscopes were implemented, providing user-friendly GUIs to prepare and perform complex experiments. Moreover, real-time, remote tracking of the ongoing acquisition, including interactive data visualization, and real-time data analysis are enabled using a file exchange-based interface between Qudi-HiM tasks and a status tracker application.

Other Python-based solutions for general control of optical microscopes exist ( Python Microscopy, Oxford Microscope-Cockpit ^27^, Tormenta ^28^, Storm-Control, MicroMator ^29^). Some of these software packages provide an out-of-the-box solution for standard acquisition modes ( *e.g.* single-molecule localization microscopy), while Qudi-HiM requires more programming skills to set up the desired functionalities and establish the necessary configuration files. However, this apparent limitation provides the required flexibility to adapt to complex, non-standard imaging modalities that require the design of complex acquisition pipelines and the control of conventional microscopy hardware components (cameras, lasers) as well as of other less standard devices ( *e.g.* for fluid control). In this paper, we provided an example of the capabilities of Qudi-HiM to implement a full sequential-imaging acquisition modality for multiplexed DNA imaging.

Qudi-HiM was initially developed on a home-made Hi-M microscope made from independent hardware components, and later deployed in a minimal amount of time to other setups, keeping the same experimental logic and user interfaces. Therefore, hardware variability remains transparent to the end-users, facilitating both training and transfer of competences. Qudi-HiM is now used on a daily basis on two Hi-M setups and a standard single-molecule localization microscope at the Center for Structural Biology (Montpellier). It is worth noting that the initial architecture of Qudi offers high flexibility and helped us design Qudi-HiM as a versatile tool for multiplexed imaging. In particular, tasks initially developed for Hi-M imaging can easily be adapted according to user requirements, and the design of new tasks is greatly facilitated, simplifying Qudi-HiM evolution alongside research projects.

## Data availability

### Underlying data

Zenodo: Minimal dataset to test multiplexed DNA imaging (Hi-M) software pipelines [dataset] https://doi.org/10.5281/zenodo.6351755 ^33^

- scan_001_RT27_001_ROI_converted_decon_ch00.tif (Fiducial image for RT27 for ROI 001 using 561-nm laser line)
- scan_001_RT27_001_ROI_converted_decon_ch01.tif (RT27 image for ROI 001 using 641-nm laser line)
- scan_001_RT29_001_ROI_converted_decon_ch00.tif (Fiducial image for RT29 for ROI 001 using 561-nm laser line)
- scan_001_RT29_001_ROI_converted_decon_ch01.tif (RT29 image for ROI 001 using 641-nm laser line)
- scan_001_RT37_001_ROI_converted_decon_ch00.tif (Fiducial image for RT37 for ROI 001 using 561-nm laser line)
- scan_001_RT37_001_ROI_converted_decon_ch01.tif (RT37 image for ROI 001 using 641-nm laser line)
- scan_006_DAPI_001_ROI_converted_decon_ch00.tif (Fiducial image for DAPI for ROI 001 using 561-nm laser line)
- scan_006_DAPI_001_ROI_converted_decon_ch01.tif (DAPI image for ROI 001 using 405-nm laser line)
- scan_006_DAPI_001_ROI_converted_decon_ch02.tif (RNA image for ROI 001 using 488-nm laser line)

Data are available under the terms of the Creative Commons Attribution 4.0 International license (CC-BY 4.0).

## Software availability

Source code available from: https://github.com/NollmannLab/qudi-HiM

Archived source code at time of publication: https://doi.org/10.5281/zenodo.6379944 ^30^

License: Creative Commons Attribution 4.0 International license (CC-BY 4.0)

Qudi-HiM has been added to the RRID database (record ID: SCR_022114).

## Ethics and consent

Ethical approval and consent were not required.
