# Supplementary material for: Qudi-HiM: an open-source acquisition software package for highly multiplexed sequential and combinatorial optical imaging
Source: Open Res Eur. 2022 Jul 19;2:46. Originally published 2022 Apr 7. [Version 2] doi: 10.12688/openreseurope.14641.2 (PMC10445908; doi:10.12688/openreseurope.14641.2)
Supplement: Peer review history

# Peer review: Reviewer response for version 2

**DOI:** 10.21956/openreseurope.16171.r29737

**Published:** 2022-08-17

**Authors:** Ricardo Henriques

**Affiliations:** 1 Instituto Gulbenkian de Ciência, Oeiras, Portugal

I'm happy with the revisions and for the paper to be indexed.

Is the rationale for developing the new software tool clearly explained?

Yes

Is the description of the software tool technically sound?

Partly

Are sufficient details of the code, methods and analysis (if applicable) provided to allow replication of the software development and its use by others?

Yes

Is sufficient information provided to allow interpretation of the expected output datasets and any results generated using the tool?

Partly

Reviewer Expertise:

NA

I confirm that I have read this submission and believe that I have an appropriate level of expertise to confirm that it is of an acceptable scientific standard.

# Peer review: Reviewer response for version 2

**DOI:** 10.21956/openreseurope.16171.r29736

**Published:** 2022-07-25

**Authors:** Romain Laine

**Affiliations:** 1 MicrographiaBio, London, UK

I have now reviewed the changes made by the authors in response to my suggestions and I am pleased that everything was addressed appropriately.

Is the rationale for developing the new software tool clearly explained?

Partly

Is the description of the software tool technically sound?

Yes

Are sufficient details of the code, methods and analysis (if applicable) provided to allow replication of the software development and its use by others?

Yes

Is sufficient information provided to allow interpretation of the expected output datasets and any results generated using the tool?

Partly

Reviewer Expertise:

NA

I confirm that I have read this submission and believe that I have an appropriate level of expertise to confirm that it is of an acceptable scientific standard.

# Peer review: Reviewer response for version 1

**DOI:** 10.21956/openreseurope.15811.r29003

**Published:** 2022-05-16

**Authors:** Romain Laine

**Affiliations:** 1 MicrographiaBio, London, UK

Barho et al. provide a great overview of their Qudi-HiM tool, designed to improve the integration of microscopy hardware components for automated imaging, especially that required by their HiM approach. It is a very timely and well organised piece of work describing the important modules and steps of a use-case large-scale acquisition pipeline.

 The tool was built with the end-user in mind and robustness which is again very important for the community increasingly shifting towards Python-based acquisition platforms, and away from more traditional ones like MicroManager and MATLAB. It clearly uses state-of-the-art computer science tools with web interfaces, efficient Qt GUI and handles to advanced image analysis pipelines. I think that the microscopy development community is interested in understanding this extension of the author’s Nature Protocol paper (Cardozo Gizzi et al., 2020).

 I only have one main suggestion to the authors about this work and it’s about placing the work in context. In my view, developing acquisition tools in this day and age needs to be subject to explicit standardization and reporting methods, essential for reproducibility and transparency as highlighted in the Nature Methods Focus 2021 ( https://www.nature.com/collections/djiciihhjh).So, although the authors describe the tool capabilities at high level and provide the code through their Github page, I would enjoy seeing a better description of the ways in which efforts were put into standardization and reporting. For instance, how easy is it to swap one piece of hardware for another? What are the necessary steps and pre-requisite? Especially in the light of the abstract hardware device class definition.

 Regarding reporting, what is reported and how can a user handle what is reported to help them understand their experiment (or failure thereof)?

 And finally, discussing the advantages and disadvantages of Qudi-HiM in light of the other Python based platforms (as cited in introduction) would be very useful.

 As a minor point, it would be useful to have a figure dedicated to the experiment configurator module, showing how the experimental pipeline is set up, highlighting the versatility of the acquisitions made possible here.

Is the rationale for developing the new software tool clearly explained?

Partly

Is the description of the software tool technically sound?

Yes

Are sufficient details of the code, methods and analysis (if applicable) provided to allow replication of the software development and its use by others?

Yes

Is sufficient information provided to allow interpretation of the expected output datasets and any results generated using the tool?

Partly

Reviewer Expertise:

I am a researcher working on developing new microscopy methods

I confirm that I have read this submission and believe that I have an appropriate level of expertise to confirm that it is of an acceptable scientific standard, however I have significant reservations, as outlined above.

# Response

**Published:** 2022-07-07

**Authors:** Marcelo NOLLMANN

We provide below a point-by-point answer to the reviewer comments. 

**Comment 1**. Barho et al. provide a great overview of their Qudi-HiM tool, designed to improve the integration of microscopy hardware components for automated imaging, especially that required by their HiM approach. It is a very timely and well organised piece of work describing the important modules and steps of a use-case large-scale acquisition pipeline. The tool was built with the end-user in mind and robustness which is again very important for the community increasingly shifting towards Python-based acquisition platforms, and away from more traditional ones like MicroManager and MATLAB. It clearly uses state-of-the-art computer science tools with web interfaces, efficient Qt GUI and handles to advanced image analysis pipelines. I think that the microscopy development community is interested in understanding this extension of the author’s Nature Protocol paper (Cardozo Gizzi et al., 2020).

**Response:** We thank the reviewer for his comments and suggestions to improve our work. Our point by point answers are provided below.  

**Comment 2**. I only have one main suggestion to the authors about this work and it’s about placing the work in context. In my view, developing acquisition tools in this day and age needs to be subject to explicit standardization and reporting methods, essential for reproducibility and transparency as highlighted in the Nature Methods Focus 2021 ( https://www.nature.com/collections/djiciihhjh).So, although the authors describe the tool capabilities at high level and provide the code through their Github page, I would enjoy seeing a better description of the ways in which efforts were put into standardization and reporting.    For instance, how easy is it to swap one piece of hardware for another? What are the necessary steps and prerequisite? Especially in the light of the abstract hardware device class definition.  

**Response**: To address this important remark, we now include a new section in the revised text providing detailed instructions on how hardware exchange is handled in Qudi-HiM:

 “Currently supported hardware and extensions: An overview of all devices that are available in the current version of Qudi-HiM is shown in Table 2. An online resource with the most up to date supported hardware is available here. Swapping devices between experimental setups is straight-forward, as long as the device has been interfaced in Qudi-HiM’s hardware modules. This simply requires updating the setup configuration file to interface the new piece of hardware. Example entries are provided in the hardware modules documentation (see examples of configuration files at: https://github.com/NollmannLab/qudi-HiM/tree/master/config/custom_config.  Extending the available hardware is achieved by setting up a new hardware module and implementing an abstract interface using existing Python APIs or C libraries. Finally, the configuration file is updated to interface the new hardware component.“

**Comment 3**. Regarding reporting, what is reported and how can a user handle what is reported to help them understand their experiment (or failure thereof)?  

**Response**; We thank the reviewer for pointing this out. Qudi-HiM reports the outcome of experiments in several ways. First, Qudi-HiM continuously reports information concerning its status and failures while the pipeline is executed. This log is displayed on the Qudi manager GUI and is very useful to monitor the experiment progress, as well as to debug the code (when developments are made). Second, Qudi-HiM exports the metadata of any ongoing experiment (e.g. sample name, exposure time, laser lines, intensity, autofocus parameters, sample XY position, etc) in a human readable format (.txt or .yml) along with or as header of the associated image file. Third, Qudi-HiM real-time operation can be monitored by connecting to a Bokeh server providing key experimental parameters (e.g. cycle number, flow rate) and real-time analysis of the acquired imaging data. Overall, these three reporting solutions are great tools for the user to monitor, diagnose, and debug any experiment.

 We have made several changes to the text to address this issue:  

 a. The following information was modified in the text to clarify how the log is working: “The current status of the experiment can be tracked in real-time using the log displayed by the Qudi manager module. Relevant information (e.g. flow rate, ROI position, etc.) are also displayed on the GUI elements that remain accessible during an experiment.”

 b. We extended the description of metadata exported by Qudi-HiM, and explained how the software can be adapted to include additional metadata: “[...] the storage of imaging data using a filename format encoding the cycle number and ROI, and of metadata containing the imaging and fluidics parameters. For Hi-M experiments, metadata saved with each image include sample name, imaging information such as exposure time, laser lines and intensity, autofocus parameters, sample XY position, scan step size and total Z range covered. To comply with a standardized metadata format ^29^ depending on the type of experiment that is performed, it is possible to add custom information by adapting the underlying software module containing the instructions of the experiment.”

 c. We added a new figure (revised Figure 4) where the information logged by Qudi-HiM are explicitly mentioned.   

**Comment 4**. And finally, discussing the advantages and disadvantages of Qudi-HiM in light of the other Python based platforms (as cited in introduction) would be very useful.  

**Response**: To address this remark, we added a dedicated subsection to the “Impact and conclusions” section where we discuss the advantages and disadvantages of Qudi-HiM compared to other Python-based microscope platforms:

 “Other Python-based solutions for general control of optical microscopes exist ( Python Microscopy, Oxford Microscope-Cockpit ^25^ , Tormenta ^26^ , Storm-Control, MicroMator ^27^ ). Some of these software packages provide an out-of-the-box solution for standard acquisition modes (e.g. single-molecule localization microscopy), while Qudi-HiM requires more programming skills to set up the desired functionalities and establish the necessary configuration files. However, this apparent limitation provides the required flexibility to adapt to complex, non-standard imaging modalities that require the design of complex acquisition pipelines and the control of conventional microscopy hardware components (cameras, lasers) as well as of other less standard devices (e.g. for fluid control). In this paper, we provided an example of the capabilities of Qudi-HiM to implement a full sequential-imaging acquisition modality for multiplexed DNA imaging.”  

**Comment 5**. As a minor point, it would be useful to have a figure dedicated to the experiment configurator module, showing how the experimental pipeline is set up, highlighting the versatility of the acquisitions made possible here.  

**Response**: We have added a figure explaining in more detail the steps to set up the experiment using the experiment configurator module and the subsequent interactions between the Qudi task runner and the experiment scripts (Figure 4 in the revised manuscript). We modified and extended the text of the Automatized experiments section as follows:

 [...] Each task requires a set of user parameters that needs to be easily accessible and adapted according to the sample and the experimental conditions. For this reason, an experiment configurator module for the setup of experiments is included in Qudi-HiM. Depending on the selected task, the configurator GUI displays a form that provides access to all required parameters and verifies their validity before saving the configuration (Figure 4). The configuration is stored as a YAML file and is subsequently loaded by the Python script that contains all information for the task execution. The script itself is controlled via the Task runner GUI: Here, the user selects which task to run and has the ability to run, stop, pause or resume the task. The task script connects to all required experimental logic modules to  combine the necessary high level hardware actions.  Since multiplexed acquisitions can last for several days, it is convenient for the users to track the current status of their experiment. Besides visualization on the Qudi-HiM GUIs and a textual information status on the log for local observation, we also provided a file exchange-based interface from Qudi-HiM software to a status-tracker application using Bokeh ( Bokeh documentation) to display key parameters of the experiment (e.g. cycle number, fluidics status, etc.) and a real-time pre-analysis of the acquired image data.”

# Peer review: Reviewer response for version 1

**DOI:** 10.21956/openreseurope.15811.r29002

**Published:** 2022-05-03

**Authors:** Ricardo Henriques, Simao Coelho

**Affiliations:** 1 Instituto Gulbenkian de Ciência, Oeiras, Portugal

Barho et al. present an open-source hardware control and software package, written in Python. The primary function is to perform basic image acquisition, some hardware control, and automation, principally for fluid handling. In practice, Qudi-HiM represents a software upgrade to an already existing package, the Hi-M.

 Previously, comparable software has been made available via Gizzi et al. 2020 amongst others. Via GitHub links (in Gizzi et al. 2020), the control software (LabVIEW) and analysis software (Matlab) have already been readily available to the public. Whilst the authors are correct that LabVIEW and Matlab have costs associated with them, many institutes mitigate this by having site licences, which allow for routine access. Although LabVIEW remains the preferred software for instrumentation control, Python developers have made significant advances.

 With the advent of Python, it is reasonable to include this upgrade and stands to benefit less fortunate research groups without site licences. The Python code, provided by the Nollmann lab GitHub page, has already become popular. This is a good indication that there is enthusiasm for this package.

 Although the software provides the simulation of dummy devices, it does not, however, state what it is currently compatible with, nor which hardware was used here. Hardware integration is barely mentioned. It is unclear which optomechanical components integrate the microscope used (lasers, filter wheels etc).

 In the ‘Use cases’ the authors state: ‘reproducible focusing of the sample for all the pre-defined ROIs (with ~50 nm accuracy)’. Does this not depend on the hardware in question? I suggest expanding this section to fully detail the hardware and describe current software limitations.

 Altogether, the software presents a valid platform with great potential but is insufficiently described in the current format.

Is the rationale for developing the new software tool clearly explained?

Yes

Is the description of the software tool technically sound?

Partly

Are sufficient details of the code, methods and analysis (if applicable) provided to allow replication of the software development and its use by others?

Yes

Is sufficient information provided to allow interpretation of the expected output datasets and any results generated using the tool?

Partly

Reviewer Expertise:

My research entails the development of optical microscopy approaches, including hardware and software.

We confirm that we have read this submission and believe that we have an appropriate level of expertise to confirm that it is of an acceptable scientific standard, however we have significant reservations, as outlined above.

# Response

**Published:** 2022-07-07

**Authors:** Marcelo NOLLMANN

We provide below a point by point response to reviewer comments.

**Comment 1: **Barho et al. present an open-source hardware control and software package, written in Python. The primary function is to perform basic image acquisition, some hardware control, and automation, principally for fluid handling. In practice, Qudi-HiM represents a software upgrade to an already existing package, the Hi-M.

 Previously, comparable software has been made available via Gizzi et al. 2020 amongst others. Via GitHub links (in Gizzi et al. 2020), the control software (LabVIEW) and analysis software (Matlab) have already been readily available to the public. Whilst the authors are correct that LabVIEW and Matlab have costs associated with them, many institutes mitigate this by having site licences, which allow for routine access. Although LabVIEW remains the preferred software for instrumentation control, Python developers have made significant advances.

 With the advent of Python, it is reasonable to include this upgrade and stands to benefit less fortunate research groups without site licences. The Python code, provided by the Nollmann lab GitHub page, has already become popular. This is a good indication that there is enthusiasm for this package.  

**Response: **We thank the reviewer for his comments and suggestions to improve our work. Our point by point answers are provided below.

**Comment 2:** Although the software provides the simulation of dummy devices, it does not, however, state what it is currently compatible with, nor which hardware was used here. Hardware integration is barely mentioned. It is unclear which optomechanical components integrate the microscope used (lasers, filter wheels etc).

**Response:** We thank the reviewer for this remark. In the documentation page for our original Github repository, we had included a section that described in detail the hardware components that are currently used for the installation of Qudi-HiM in four different microscopy setups: https://github.com/NollmannLab/qudi-HiM/blob/master/documentation/qudi-cbs%20documentation/qudi-HiM_hardware/overview_hardware_qudi-him.md. In addition, we added a new table (Table 2) with the instruments currently controllable by Qudi-HiM. These two resources, in addition to a new section describing the steps required to add new hardware components (see below), are now mentioned. The revised manuscript is as follows:

 “Currently supported hardware and extensions An overview of all devices that are available in the current version of Qudi-HiM is shown in Table 2. An online resource with the most up to date supported hardware is available here. Swapping devices between experimental setups is straight-forward, as long as the device has been interfaced in Qudi-HiM’s hardware modules. This simply requires updating the setup configuration file to interface the new piece of hardware. Example entries are provided in the hardware modules documentation (see examples of configuration files here:  https://github.com/NollmannLab/qudi-HiM/tree/master/config/custom_config).  Extending the available hardware is achieved by setting up a new hardware module and implementing an abstract interface using existing Python APIs or C libraries. Finally, the configuration file is updated to interface the new hardware component.“

**Comment 3**. In the ‘Use cases’ the authors state: ‘reproducible focusing of the sample for all the pre-defined ROIs (with ~50 nm accuracy)’. Does this not depend on the hardware in question? I suggest expanding this section to fully detail the hardware and describe current software limitations.

**Response**: The reviewer’s comment is correct. This 50 nm precision is related to the optical design of our autofocus system as well as the instruments we are using. Qudi-HiM is only a means to synchronize these devices to perform calibration and focus stabilization in real time. For clarity, we removed the mention of the autofocus precision from the text and added a reference to  publications that already thoroughly describe the hardware components and performance of the autofocus system. The revised manuscript is as follows:

 “[...] (2) the correct and reproducible focusing of the sample for all the pre-defined ROIs (see ^5,10^ for a detailed description of the autofocus hardware) [...]”

**Comment 4**. Altogether, the software presents a valid platform with great potential but is insufficiently described in the current format.

**Response: **We expect that the changes added to the revised manuscript would have provided enough detail to satisfy this comment of the reviewer.
